# The role of P53 up-regulated modulator of apoptosis (PUMA) in ovarian development, cardiovascular and neurodegenerative diseases

**DOI:** 10.1007/s10495-021-01667-z

**Published:** 2021-03-30

**Authors:** Mei Li

**Affiliations:** Department of Animal Genetics, Breeding and Reproduction, College of Animal Science and Technology, Nanjing Agricultural University, 1 Weigang, Nanjing, 210095 China

**Keywords:** PUMA, Apoptosis, Excessive cell death, Ovary, Cardiovascular disease, Neurodegenerative disease

## Abstract

P53 up-regulated modulator of apoptosis (PUMA), a pro-apoptotic BCL-2 homology 3 (BH3)-only member of the BCL-2 family, is a direct transcriptional target of P53 that elicits mitochondrial apoptosis under treatment with radiation and chemotherapy. It also induces excessive apoptosis in cardiovascular and/or neurodegenerative diseases. PUMA has been found to play a critical role in ovarian apoptosis. In the present paper, we review the progress of the study in PUMA over the past two decades in terms of its inducement and/or amplification of programmed cell death and describe recent updates to the understanding of both P53-dependent and P53-independent PUMA-mediated apoptotic pathways that are implicated in physiology and pathology, including the development of the ovary and cardiovascular and neurodegenerative diseases. We propose that PUMA may be a key regulator during ovary development, provide a model for PUMA-mediated apoptotic pathways, including intrinsic and extrinsic apoptotic pathways.

## Introduction

Apoptosis is a morphologically and biochemically distinct form of cell death that occurs in physiology and pathology, including ovarian development and cardiovascular and neurodegenerative diseases [1, 2]. It is characterized by cell shrinkage, membrane blebbing, DNA fragmentation, chromatin condensation, and apoptotic body formation [3]. It is usually initiated by either death receptor pathway or through the mitochondrial pathway, regulated by the BCL-2 family of proteins. The BCL-2 proteins consist of pro-survival and pro-apoptotic members. The careful modulation of the balance between these two groups of BCL-2 proteins can largely determine a cell’s fate between life and death.

PUMA, also known as BCL-2 binding component 3 (BBC3), a BCL-2 homology 3 (BH3)-only protein of the BCL-2 family, was originally identified as a P53-downstream target, independently discovered by three separate groups [4–6]. PUMA binds to all of the anti-apoptotic BCL-2 members and inhibits their pro-survival activity, and it can also directly activate the pro-apoptotic effectors BCL-2-associated X protein (BAX) and BCL-2 antagonist/killer (BAK) to cause mitochondrial outer membrane permeabilization (MOMP), resulting in the release of apoptogenic molecules, including second mitochondria-derived activator of caspases (SMAC), serine protease OMI, and cytochrome c from the mitochondrial intermembrane space into the cytoplasm. Cytochrome c binds apoptotic protease-activating factor 1 (APAF 1) in the cytosol to form the apoptosome to activate caspase-activity cascades and cell apoptosis in various cell types [7]. In this present paper, we review the progress in the study of PUMA in relation to its inducement and/or amplification of programmed cell death over the past two decades. We also describe improvements to the understanding of PUMA-mediated signaling pathways, summarize the role of PUMA in ovarian development, and in cardiovascular and neurodegenerative diseases, and propose a model for PUMA-mediated apoptosis.

## Apoptotic pathways

**Fig. 1:**
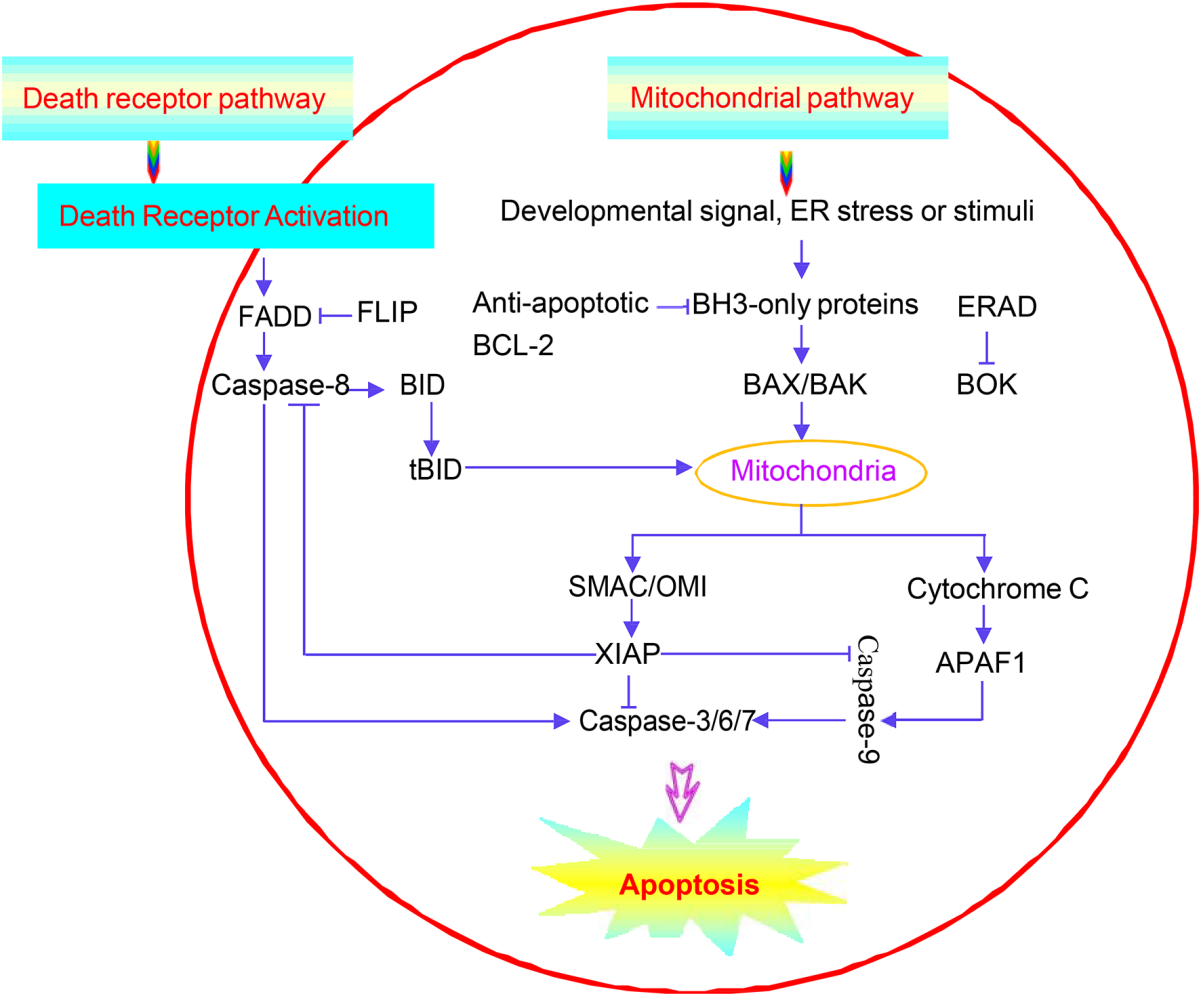
Apoptosis pathways. The death receptor and mitochondrial pathways of apoptosis are shown. The mitochondrial pathway is activated by developmental cues, the endoplasmic reticulum, or other stimuli. These stimuli trigger the BH3-only proteins, which then inhibit the BCL-2-like pro-survival proteins. The inhibition of the BCL-2-like pro-survival proteins leads to the activation of BCL-2-associated X protein (BAX) and/or BCL-2 antagonist/killer (BAK). Activated BAX or BAK oligomerizes and forms pores to cause mitochondrial outer membrane permeabilization (MOMP), resulting in the release of apoptogenic molecules, including second mitochondria-derived activator of caspases (SMAC), serine protease OMI, and cytochrome c from the mitochondrial intermembrane space into the cytoplasm. Cytochrome c binds apoptotic protease-activating factor 1 (APAF 1) in the cytosol to form the apoptosome, which serves as a platform for the activation of caspase-9. Caspase-9 then activates the effector caspases (caspase-3, caspase-6, and caspase-7), which leads to cell demolition. Caspase activation can be blocked by the X-linked inhibitor of apoptosis protein (XIAP), which in turn is inhibited by the released SMAC and OMI proteins from the mitochondria. The death receptor pathway is activated when the ligands of the tumor necrosis factor family bind to their respective death receptors on the cell membrane. This results in cleavage of caspase-8, leading to the activation of effector caspases (caspase-3, caspase-6, and caspase-7) and cellular destruction. Death receptor signaling can also result in BID cleavage by caspase-8, leading to the generation of active tBID, which then engages the mitochondrial pathway

Mammals have two distinct but ultimately convergent pathways to apoptosis [8]: the death-receptor (also called extrinsic) pathway and the BCL-2-regulated (also called intrinsic or mitochondrial) pathway [9–11] (Fig. 1). The death receptor pathway is induced by death ligands and their cognate-death receptor, the adapter molecule FAS-associated death domain (FADD) or TNF receptor-associated protein with the death domain, which form the death-inducing signal complex (DISC), cause the recruitment and activation of initiator caspase-8 followed by activation of the executioner caspases-3, -6, and -7 [7, 12]. The inhibition of this death-receptor-induced apoptosis can be mediated by FADD-like ICE inhibitory protein (FLIP), which competitively blocks the processing of pro-caspase-8 at the DISC and keeps cells healthy [7, 12].

By contrast, the mitochondrial pathway is triggered by cellular stress, developmental cues, and endoplasmic reticulum (ER) stress [13]. The BCL-2 protein family contains the pro-apoptotic members, such as BID, BIM, PUMA, BAX, and BAK, and the antiapoptotic members, such as BCL-2, BCL-XL, and MCL-1. BAX, BAK, and BCL-2-related ovarian killer protein (BOK) directly cause MOMP, resulting in the release of apoptogenic molecules, including SMAC (also known as DIABLO), serine protease OMI (also known as HTRA2), and cytochrome c. Active BAX and BAK are inhibited by anti-apoptotic BCL-2 proteins. The BH3-only proteins in the BCL-2 family inhibit the anti-apoptotic BCL-2 proteins and thus unleash BAX and BAK from their restraint by the pro-survival BCL-2 family members to affect MOMP. Cytochrome c, which is released upon MOMP from the inter-mitochondrial space into the cytosol, binds to the cytosolic APAF1 protein and triggers apoptosome formation to activate initiator caspase-9, which then activates the executioner caspases-3, -6, and -7. Caspase-8 can proteolytically activate the BH3-only protein, BID, and by this means cause MOMP by activating BAX and BAK. Caspase activation can be blocked by X-linked inhibitor of apoptosis protein (XIAP), members of inhibitor of apoptosis proteins (IAPs), which in turn are inhibited by the SMAC and OMI proteins released from the mitochondria [7, 12]. Several proteins that are regulated by cleavage by effector caspases affect the distribution of phospholipids in the plasma membrane to allow phosphatidylserine (PS), which is normally constrained to the inner leaflet, to be exposed on the cell surface. The exposure of PS is a signal that promotes the phagocytosis of a dying cell prior to the loss of plasma membrane integrity [12].

## PUMA

### Basic information

**Fig. 2:**
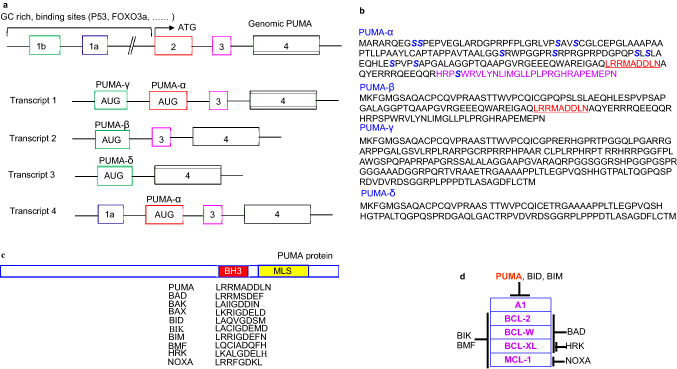
PUMA information. **a** Genomic structure of PUMA and alternative transcripts. **b** Amino acid sequence of PUMA-α, -β, -γ, and -δ. Conserved serines (S), BH3, and C-terminal domains in the amino acid sequence of PUMA are shown. S is given in blue, the BH3 domain is in red, and the C-terminal domain in purple. **c** PUMA protein and two functional domains, namely, the BH3 and C-terminal mitochondria-localization signal (MLS) domains. The BH3 domain in PUMA is compared to the other pro-apoptotic BCL-2 family members. **d** PUMA and the other BH3-only proteins have different binding profiles to the anti-apoptotic BCL-2 protein (Color figure online)

PUMA is a member of a subfamily of BH3-only pro-apoptotic proteins. It is highly conserved between human and mouse, with an over 90% sequence identity at both the DNA and protein levels. The genomic structure of PUMA is also similar between human and mouse [5, 6]. The human PUMA gene contains three coding exons (2–4) and two noncoding exons (1a and b), all of which (except for exon 1b) are conserved in mouse. PUMA has four transcripts (α, β, γ, and δ), and the length of the PUMA transcript is about 1.6–1.9 kb. Extensive alternative splicings result in multiple PUMA transcript variants [5, 6]. Only PUMA-α and -β encoded proteins with the BH3 domain display pro-apoptotic activity, and they interact with members of the BCL-2 family in the mitochondrial membrane (Fig. 2).

The BH3 domain of PUMA forms an amphipathic α-helical structure, which is required for it to directly interact with BCL-2-like proteins in the mitochondrial membrane to drive cytochrome c relocation from the mitochondria to the cytoplasm and activate procaspases-9 and -3 [5, 6]. PUMA also has a functional domain of the mitochondrial localization signal (MLS), which is localized in the C-terminal region of the molecule as a hydrophobic domain. The MLS directs the mitochondrial localization of PUMA [14]. The BH3 domain and MLS are essential for the ability of PUMA to induce apoptosis (Fig. 2).

The expression of PUMA is very low in normal cells, and cytosolic PUMA is usually undetectable. The high guanine and cytosine content of PUMA promoter at exon 1a and intron 1 favors the formation of secondary structures that limit the accessibility of the transcriptional machinery or recruit transcriptional repressors or chromatin-modifying proteins to prevent active transcription and maintain low basal expression levels in unstressed cells [6] (Fig. 2). Low PUMA expression is also associated with post-translational modification of PUMA. PUMA is regulated by phosphorylation on Serine (Ser) residues in multiple sites, such as 9, 10, 36, 96, 106, and 166 through post-translational modification [15]. Ser 10 is the major site for PUMA phosphorylation, and it regulates PUMA degradation by association with heat shock 70 kDa protein 8, leading to its lysosome translocation and uptake through chaperone-mediated autophagy, thus inhibiting PUMA-induced apoptosis [16]. However, the phosphorylation of PUMA at Ser 96 and Ser 106 is required for the interaction of PUMA with mitochondrial pyruvate carrier (MPC) to disrupt mitochondrial pyruvate uptake, leading to prime pathology [17, 18].

PUMA can be induced by many transcription factors that bind to it at the exon 1 and intron 1 regions of its promoter (Fig. 2). The function of P53 is best understood in relation to the transcription factors that activate PUMA [14, 19]. The P65 or P52 subunit of nuclear factor-κB (NF-κB) can facilitate P53-dependent PUMA induction through P53-dependent recruitment to PUMA promoter following certain forms of DNA damage [19]. P63 and P73, which are members of the P53 family, also activate PUMA transcriptionally [19, 20]. In addition to P53, several other transcription factors are implicated in PUMA induction. Forkhead box O (FOXO) family members FOXO1 and FOXO3a mediate PUMA induction [21–23]. CAAT/enhancer-binding protein (C/EBP) homologous protein (CHOP, also known as DNA damage inducible transcript 3, growth arrest- and DNA damage-inducible gene 153), E2 transcription factor (E2F1), TRIB3/TRB3 (Tribbles homolog 3), and activator protein 1 (AP-1/c-Jun) are involved in PUMA induction through ER stress [14, 24, 25]. Moreover, other transcriptional factors are involved as well, including C/EBP-β, cAMP response element binding protein (CREB), nuclear factor of activated T cells (NFAT), specificity protein 1 (SP1), c-MYC, interferon (IFN) regulatory factor 1(IRF-1), Caenorhabditis elegans SMA and Drosophila mothers against decapentaplegic homolog 4 (SMAD4) [14, 26, 27]. PUMA can also be negatively regulated by transcriptional repressors, such as SLUG, certain alternative splice products of P73 or P63, and microRNAs [19, 28, 29].

PUMA is expressed and induced by a range of stimuli, including genotoxic stress, hypoxia, ER stress, mitochondrial perturbation, deregulated oncogene expression, toxins, growth factor/cytokine withdrawal, altered redox status, and infection in different cell types of humans and mice [14, 19]. PUMA functions in either a P53-dependent or P53-independent apoptotic manner. Once expressed, PUMA binds to all of the anti-apoptotic BCL-2 members proteins (Fig. 2) and also directly activates the pro-apoptotic effectors BAX and BAK, causing MOMP, caspase cascades and cell apoptosis in various cell types [7, 12]. PUMA can also bind to apoptosis repressors with caspase recruitment domain (ARC) to activate caspase-8, which cleaves BID into tBID and accelerates BAX mitochondrial translocation in the heart and brain [30–32]. It should be noted that PUMA links intrinsic and extrinsic pathways through ARC, which can be P53-dependent or P53-independent (FOXO3a) [30–32]. Reactive oxygen species (ROS) can also upregulate PUMA expression in vitro and in vivo [32, 33]. ROS, caspases, cytochrome c, and other signalers participate in positive feed-forward loops to amplify cell death, as is implicated in physiology and pathology.

### PUMA paradox

The PUMA gene is a double-edged sword. It has been shown that PUMA upregulation is not always linked to apoptosis. Monocytes that are stimulated by pro-inflammatory cytokines can promote PUMA upregulation in the endothelial cells of the human umbilical vein. However, elevated PUMA protein levels do not result in apoptosis in cells. PUMA is highly expressed during cell proliferation and survival in vascular and microglia cells through the regulation of autophagy [34]. PUMA-mediated autophagy is either cytoprotective or cytotoxic [35–37]. PUMA upregulation inhibits mitochondrial pyruvate uptake and oxidative phosphorylation, and it increases glycolysis in hepatocellular carcinoma, which depends on IκB kinase-mediated phosphorylation of PUMA at Ser 96/106 [17]. PUMA is also overexpressed in many other human cancers [17, 34], and the loss of PUMA ablates tumorigenesis in certain mouse models [38, 39]. Thus, PUMA is also an oncogene [17, 38, 39]. Furthermore, the genetic ablation or inhibition of PUMA leads paradoxically to protective effects of cells and hematopoietic stem/progenitor cells in mouse intestinal and hematopoietic systems [38–40]. It should be noted that the PUMA paradox has been posited as a riddle that requires future study [12]. In addition, PUMA inhibitors (PUMAi) are designed to inhibit PUMA-dependent and radiation-induced apoptosis in vitro and to prevent or mitigate intestinal damage and apoptosis induced by inflammatory cytokines, ROS, or chemotherapy [41]. PUMAi have also been confirmed by biochemical assays, including GST-pull down assay and fluorescence polarization (FP) assay, to effectively disrupt the interaction between PUMA and BCL-XL (unpublished data). PUMAi protect against chemotherapy-induced intestinal injury [42]. All of the PUMAi have recently been reviewed [43].

## PUMA-mediated signaling pathways

### P53-dependent pathways

PUMA can be transcriptionally activated by P53 in vitro and in vivo [14, 19]. Mouse double minute 2 (MDM2) is a P53-negative regulator, and MDM2 inhibitor (Nutlin-3a) kills hematological cancer-derived cell lines in vitro and in mice, mostly through the P53-mediated induction of PUMA [44]. P53 transcriptionally represses ARC expression but activates PUMA expression, and PUMA competitively binds to ARC with caspase-8, which cleaves BID into tBID to accelerate BAX translocation in heart and brain [30–32]. The P53-induced PUMA apoptotic program can be interfered with by Scratch2 (SNAI2), a zinc finger transcriptional repressor of the SLUG/SNAIL family [28]. In addition, SLUG (SNAI2) directly binds PUMA at intron 1 and represses its expression [29]. It is noteworthy that PUMA can disrupt the interaction between cytosolic P53 and BCL-XL, allowing P53 to promote DNA damage-induced apoptosis via direct activation of the BCL-2 effector molecules, BAX and BAK [45]. It has recently been found that the wild-type P53 suppresses pyruvate-driven oxidative phosphorylation by inducing PUMA, which inhibits mitochondrial pyruvate uptake through PUMA-MPC interaction in hepatocarcinoma [17].

PUMA can be activated through P53 post-translational modification. Lys120 acetylation in P53 mediates the acetylation of histone H4 at the PUMA promoter, promoting PUMA expression during DNA damage [46]. However, Lys382 monomethylation inhibits P53 from recruiting to PUMA promoters [47]. In addition, the cofactors of the apoptosis stimulation of of P53 protein 1/2 (ASPP1/2) and apoptosis-antagonizing transcription factor (AATF) also bind P53 at PUMA promoters and influence PUMA expression [48].

The other members of the P53 family, P73 and P63, may also activate PUMA. P63 triggers apoptosis through the transcriptional induction of PUMA [20]. P73 can regulate PUMA expression independent of P53 by binding to the same P53-responsive elements in the PUMA promoter in response to a variety of stimuli [19]. P73 and E2F1 transactivate PUMA directly by binding and activating the promoter [14]. E2F1 also transactivates P73, and this may amplify the induction of PUMA [49]. The ΔNp73 isoforms may repress the PUMA-BAX system and inhibit both TAp73- and P53-induced apoptosis [19].

### P53-independent pathways

#### JAK-STAT

The Janus kinase-signal transducer and activator of transcription (JAK-STAT) signaling pathway is involved in many crucial biological processes, such as apoptosis. PUMA is upregulated through the JAK1-STAT1 pathway to induce apoptosis for therapy in cutaneous T-cell lymphoma cell lines [50]. PUMA is also regulated by STAT3, thus inducing apoptosis in human mucoepidermoid carcinoma cell lines [51]. The precise role of PUMA in the JAK-STAT pathway awaits further study.

#### PI3K-AKT-FOXO1/3a

PUMA is a downstream target of FOXO1 and FOXO3a [21–23]. The activation of PI3K-mediated protein kinase B (PKB/AKT) results in the direct phosphorylation and cytoplasmic retention of the transcription factors FOXO1 and FOXO3a and prevents PUMA upregulation [21–23]. Serum and glutocorticoid-induced kinase 1 (SGK1) phosphorylates FOXO3a and causes it to translocate out of the nucleus, thus inhibiting PUMA expression [52]. MYC and PI3K-AKT signaling cooperatively repress FOXO3a-dependent PUMA expression [53]. However, chromatin remodeler Brg-associated factor 57 (BAF57), a subunit of SWItch/sucrose non-fermentable (SWI/SNF), executes neuron death in FoxO3a-mediated PUMA expression in cellular models of Parkinson’s disease (PD) [54]. In addition, autophagy inhibition increases the levels of FOXO3a transcription factor, and promotes PUMA upregulation, thereby increasing apoptosis [55]. Glycogen synthase kinase-3β (GSK-3β) also regulates PUMA expression, and GSK-3 suppression prevents PUMA induction by FOXO3a and P53 on growth factor withdrawal [56].

#### cAMP-PKA-CREB

cAMP is a well-characterized intracellular second messenger and plays a critical role in many biological processes. PUMA regulates the cAMP/protein kinase A (PKA)-induced apoptotic pathway in a P53-dependent manner [57]. PUMA can be transcriptionally repressed by cAMP-exchange protein activated by cAMP (Epac) signaling pathway [58]. PUMA promoter contains CREB binding sites [26]. The relationship between PUMA and CREB remains to be established.

#### MAPK

The mitogen-activated protein kinase (MAPK) pathway contains P38, c-Jun N-terminal kinase (JNK), and extracellular signal regulated kinase (ERK). PUMA is a direct transcriptional target of c-Jun, a subunit of the AP-1 complex. During activation in ER stress, PUMA expression is regulated by IRE1-JNK/c-jun [59]. PUMA induction is also dependent on P38 kinase in SH-SY5Y neuroblastoma cells [60]. It is also induced by doxorubicin through P53 and ERK1/2 pathways leading to apoptosis [61]. In addition, DNA lesions are induced by ROS or directly activated by PUMA through the P53, ERK1/2, and NF-κB pathway [61]. Oxidative stress activates JNK-P53-PUMA signaling and induces the apoptosis of granulosa cells in the ovary [62, 63].

### WNT signaling

Wingless-type mouse mammary tumor virus integration site family (WNT)-β-catenin signaling plays a key role in the development. GSK3β, a component of the WNT-β-catenin pathway, is required for the P53-mediated induction of PUMA [64]. WNT-β-catenin signaling potentially regulates follicular development negatively through FOXO3a-mediated PUMA pathway promoting granulosa cell apoptosis [65], and WNT3a-treated granulosa cells stop development through the FOXO3a-mediated PUMA pathway [66]. β-catenin may suppress PUMA induction until it is inactivated by GSK-3β [67].

### Hippo signaling

The Hippo signaling pathway regulates a range of physiological processes. Human mammalian sterile 20-like kinase 1 (MST1) is a core member of the Hippo pathway, and Yes-associated protein (YAP) is a major downstream effector molecule for the Hippo signaling pathway and a transcriptional coactivator of cell proliferation and apoptosis. PUMA can be upregulated by YAP1, which translocates to the nucleus and associates with P73, resulting in PUMA upregulation for apoptosis [68]. MST1 promotes apoptosis through the upregulation of the pro-apoptotic proteins P73, P53, PUMA, caspase-3, and YAP [69]. In addition, MST activates FOXOs [70], YAP regulates ER stress [71], and PUMA participates in ER stress. The role of PUMA in ER stress associated with Hippo signaling thus requires further study.

### TGF-β signaling

Transforming growth factor β (TGF-β) is a superfamily that regulates fundamental cellular properties and vital cellular processes, such as proliferation, differentiation, communication, apoptosis, and tissue remodeling. PUMA is a direct target of TGF-β signaling in B-cells, which mediates rapid induction of apoptosis [27]. In response to TGF-β, PUMA promoter signaling is most likely dependent on SMAD binding at positions -1923 to -1885 of the SMAD-binding region [27]. SMAD4 induces PUMA-mediated cell death through P21-activated kinase 1 suppression [72]. The P53 mediated PUMA and TGF-β signaling pathways are both essential for doxorubicin-induced cytotoxicity [73]. In addition, the Hippo pathway functions as a SMAD partner in transcriptional activation [74]. Hippo signaling also promotes the formation of the β-catenin destruction complex of WNT signaling through the phosphorylation of YAP1 and tafazzin (TAZ). P53 and its family relatives P63 and P73 are associated with WNT input [74]. SLUG inhibits PUMA and promotes cell survival [29]. Thus, the relationships among PUMA, P53/63/73, SLUG/SNAIL, TGF-β-SMAD4, WNT-β-catenin, and Hippo signaling need additional investigation.

### BCL-2 family

**Fig. 3:**
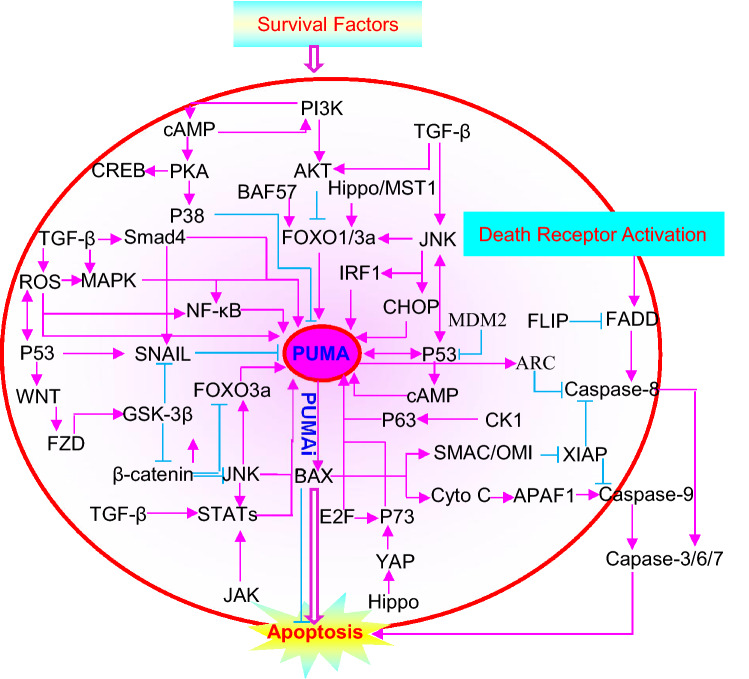
Proposed model for PUMA-mediated apoptosis. PUMA (P53 upregulated modulator of apoptosis, also known as BCL-2 binding component 3), is downstream of multiple signaling pathways. PUMA-mediated apoptosis pathways are regulated by transcriptional factors, a balance of survival and apoptotic factors, the cell–cell interactions, and communication between the ER and mitochondria. PUMA inhibition may be beneficial for therapies in some human diseases and for improving animal litter size. Activation is shown in pink, and inhibition is given in blue (Color figure online)

Several BH3-only proteins have been shown to have the canonical pro-apoptotic activity of BCL-2 family proteins, including BCL2L11/BIM, BID, BAD, BIK/NBK, BCL-2-modifying factor (BMF), activator of apoptosis hara-kiri (HRK), NOXA, BOK, NIX/BNip3L, BCL-2/adenovirus E1B 19-kD protein–interacting protein 3(BNIP3), BNIP3L, and PUMA [75]. BH3-only proteins likely have overlapping, additive, and complementary roles in ER-induced apoptosis [76]. PUMA may co-operate with other BH3-only proteins such as BIM and BID to promote its own activation and mediate the full apoptotic response [77]. Different BH3 proteins may have distinct effects, depending on their subcellular localization and the intensity of the given stimuli [78]. The relationship between PUMA and the other BH3-only proteins for inducing apoptosis is thus worth investigating. All PUMA-mediated signal pathways are proposed in Fig. 3.

## PUMA plays a critical role in the apoptotic process in an ER- and mitochondria- dependent manner

PUMA-induced apoptosis, which is linked to ER and mitochondria, plays a key role in physiology and pathology. In this paper, the role of PUMA in ovarian, cardiovascular, and neurodegenerative diseases is examined.

## PUMA may be a key regulator in ovarian development

**Fig. 4:**
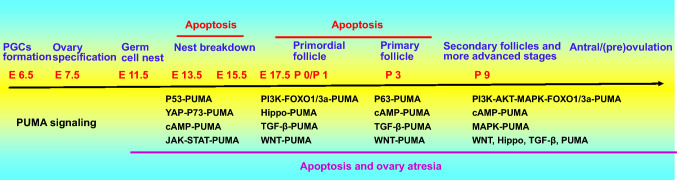
PUMA-dependent signaling pathways at different stages during ovarian development. In mice, two waves of germ cell loss due to cell death occur at embryonic days (E) 13.5–15.5 and E17.5–to postnatal day 9. PUMA-mediated possible signaling pathways are proposed at different stages during ovarian development

The mammalian ovary is the female organ for the reproductive function. In development, the ovary passes through the stages of primordial germ cell formation, germ cell nesting, nest breakdown, primordial follicles, secondary follicles, and more advanced stages, until ovulation. The total number of ovulations is important for reproductive efficiency in humans and farm animals. During ovarian folliculogenesis, over 99% of follicles undergo degeneration through follicular atresia, which shows many hallmark features of apoptotic cell death at various stages of follicular development. Many factors and signal pathways, including the P53 family, the PI3K-AKT-FOXO1/3a pathway, the cAMP-PKA-CREB pathway, the JAK-STAT pathway, Hippo signaling, TGF-β signaling, Notch signaling, and WNT-β-catenin pathway, affect the apoptosis of oocytes, granulosa cells, theca cells, and stromal cells during ovarian development [79]. In mice, two waves of cell death in germ cell loss are seen at embryonic days (E) 13.5–15.5 and E17.5 – to postnatal day 9 (Fig. 4).

## PUMA induces apoptosis in the oocytes

PUMA plays a critical role in the apoptosis of primordial germ cells in mice prior to meiotic entry, either during migration to the gonad or soon after arrival in the ovary [80]. A 2.2-fold increase in the number of germ cells in *PUMA-deficient mice* is maintained throughout ovarian development to E13.5 and results in a 1.9-fold increase in the number of primordial follicles initially established in the ovary compared to wild type mice [80]. PUMA is also involved in germ cell apoptosis and nest breakdown in cultured whole embryonic rat ovaries [81].

In addition, the loss of PUMA alone or PUMA and NOXA together protects *C57BL/6* mice from DNA damage-induced irradiation- and TAp63-mediated primordial follicle oocyte apoptosis at postnatal day 5 [20]. It is noteworthy that the loss of PUMA alone rescues 100% of the ovarian reserve following drug treatment with cyclophosphamide and cisplatin. However, the transcriptional activation pathways for PUMA may differ in response to these two drugs. Cisplatin activates a TAp63-dependent process that requires phosphorylation by both priming kinase checkpoint kinase 2 (CHK2) and executioner kinase of casein kinase 1 (CK1) in primordial mouse follicles, while cyclophosphamide acts via a TAp63-independent and FOXO3a-dependent pathway [82]. The inhibition of either of CK1 and CHK2 as well as upstream kinase ataxia telangiectasia mutated (ATM) saves oocytes in mouse ovaries from apoptosis [83]. Here, tauroursodeoxycholic acid (TUDCA), a selective inhibitor of ER stress that has been approved by the Food and Drug Administration for clinical use, inhibits granulosa cell apoptosis and promotes oocyte maturation [84, 85]. TUDCA also limits apoptosis by decreasing palmitate-induced JNK phosphorylation, PUMA upregulation and BAX activation [86]. Thus, the role of TUDCA in PUMA-mediated ovarian apoptosis should have further study.

## PUMA triggers apoptosis in somatic cells

PUMA is clearly involved in oxidative stress-induced apoptosis [22]. PUMA can be upregulated by JNK in a FOXO1-dependent manner, and JNK inhibitor (SP600125) can inhibit PUMA expression in the ovary [22]. The JNK potentiated, AKT-mediated FOXO3a, and the JNK-mediated c-Jun pathways cooperatively trigger PUMA expression in ovarian cancer cells [87]. JNK inhibitor also partially rescues PUMA-induced decreases in BCL-XL and MCL-1 in ovarian cancer cells [63]. Oxidative stress activates JNK-P53-PUMA signaling and induces apoptosis in granulosa cells [62, 63]. PUMA expression is also inhibited by P53 inhibitor (Pifithrin-α) [62] or PUMAi (unpublished data). Other hormones and small molecules, such as follicle-stimulating hormone, may also protect PUMA-mediated apoptosis through the PI3K-AKT pathway in mouse granulosa cells [88]. PUMA is upregulated in cattle granulosa cells after the treatment of fibroblast growth factor (FGF) 2 and FGF18 in a context-dependent manner [89].

In addition, oxidative stress-induced upregulation of PUMA is found in vivo through treatment with 3 nitropropionic acid (3-NP) in mice [22]. It is important here that the ovary-expression profiles in *PUMA-deficient mice* treated with 3-NP cluster at first with the wild-type (WT) mouse without treatment, and then they cluster with the treated WT mouse at 6 weeks for 7-day treatment of 3-NP by RNA-Seq (unpublished data). This suggests that a PUMA deficiency protects the mouse ovary from oxidative stress at the transcriptional profiles.

PUMA induces mitochondrial ROS generation through functional BAX, irrespective of their P53 status, and it activates nuclear factor erythroid 2-related factor 2 pathway [89], which in turn results in DNA damage response, including ATM, ATR, DNA-PKcs, CHK1, and CHK2, along with JNK activation, finally producing apoptosis in ovarian cancer cells [89]. N-acetyl-L-cysteine partially abrogates PUMA-induced apoptosis [89].

Due to the process of ovarian development and the role that PUMA plays in physiology and pathology, we propose that PUMA is a key regulator in the ovarian development, particularly where there are a range of stimuli (Figs. 3 and 4).

## PUMA inhibition is a potential therapeutic target for ameliorating cardiovascular diseases

PUMA induction through ER stress in cardiac myocytes is partially a P53-independent and partially a P53-dependent mechanism [76, 90–92]. CHOP-mediated PUMA signaling is a major component for ER stress in heart failure resulting from neonatal cardiomyocyte apoptosis and diabetic cardiomyopathy, as well as cardiac myocyte dysfunction and injury [92]. The administration of valsartan (a selective angiotensin II [Ang II] types 1 receptor antagonist) can block the activation of CHOP-mediated PUMA signaling [92]. PERK-eIF2α-CHOP-PUMA activation is also responsible for heat stress induced cell death in cardiac myocyte dysfunction and injury in mice [93]. Additionally, the administration of ursolic acid prevents heat-stress-induced cellular damage and cell death by restoring intracellular redox status and upregulating the anti-apoptotic MCL-1 protein, which, in turn, abolishes CHOP-activated PUMA induction in mouse cardiac myocytes [93]. The PUMA expression induced by the stretch in cardiomyocytes is mediated by JNK and IRF-1and induced by IFN-γ [26]. The nuclear protein 1 (NUPR1)-CHOP-P53-PUMA pathway (as well as synergy with Beclin-1) is also engaged during methamphetamine-induced cardiovascular apoptosis [94]. In addition, PUMA interacts with ARC, thereby releasing caspase -8 and inducing apoptosis in cardiomyocytes [30, 31].

In addition, doxorubicin induces acute and chronic cardiomyocyte apoptosis leading to cardiac dysfunction, cardiomyopathy, and eventually to severe heart failure and death due to activation of ERK1/2-mediated P53 and ROS-mediated MAPK and NF-κB, as well as P53-dependent PUMA upregulation signaling [61, 65]. PUMA is also regulated by P53-independent NF-κB upon ER stress, and NF-κB-dependent PUMA upregulation is indispensable for H2O2-induced cell apoptosis [65]. Propofol (50 μM) pretreatment significantly decreases H2O2-induced NF-κB activity and PUMA expression [61]. However, doxorubicin-induced cell apoptosis can also achieved through P53-dependent PUMA upregulation in H9c2 cardiacmyocytes [65].

Thus, PUMA is critical for ER-stress-induced apoptosis associated with ATP depletion, acidosis, and abnormal ER/sarcoplasmic reticulum Ca^2+^ handling in cardiac myocytes [95, 96]. It should be noted that infarct sizes and apoptotic indexes in PUMA-deficient hearts are greatly reduced under ischemia–reperfusion condition, and PUMA inhibition may be useful for treating cardiac infarcts or preventing heart failure [76, 90, 92, 95, 96].

## PUMA deficiency significantly protects neurons from ER-stress-induced apoptosis for neurodegenerative diseases

Gene expression profiling shows that PUMA alone is sufficient to induce apoptosis with tunicamycin in a P53-independent manner in human neuroblastoma cells [90]. During activation by ER stress, PUMA expression is regulated by IRE1-JNK-c-jun [59, 67], the ATF4-CHOP-PUMA signaling axis [97], and the P38-PUMA-BAX pathway, induced by 6-hydroxydopamine (6-OHDA) in PD and the subsequent activation of caspase -3 and cytochrome c release in SH-SY5Y neuroblastoma cells [60]. GSK3 regulates ER-stress-induced CHOP expression in neuronal cells [98]. GSK-3β also regulates PUMA expression [56]. TRIB3/TRB3 (a target of CHOP) is induced later than CHOP during ER stress [98], and it can promote PUMA expression in a FOXO3a-dependent manner through the dephosphorylation of AKT in PC-12 cells [98]. BAF57 executes neuron death in FoxO3a-mediated PUMA expression in cellular models of PD [54].

Multiple pathways can work together to trigger PUMA expression in the brain. The JNK and PI3K-AKT-GSK3β pathways converge to regulate FOXO3a-mediated PUMA activation, which in turn promotes BAX activation, cytochrome c release, and caspase activation, leading to neuronal cell death [67]. CHOP potentially cooperates with PI3K-AKT-FOXO3a in neuronal cells to regulate PUMA expression in response to ER stress [77]. However, insulin-like growth factor-1 effectively protects PC-12 neuronal cells from ER-stress-induced apoptosis through the PI3K-AKT and P38 MAPK pathways induced by tunicamycin, thus inhibiting PUMA expression [99]. In addition, the direct inhibition of PI3K-AKT is sufficient to induce GSK3β-dephosphorylation or activation in cerebellar granule neurons (CGNs). Decreased expression of GSK-3β activates pro-survival WNT-β-catenin signaling [100]. NFAT is likely to be a repressor of PUMA that is removed upon GSK-3β activation in CGNs [67]. In addition, NFAT is a key regulator of cell survival and death, depending on the partner that NFAT interacts with [101]. Thus, the interaction between PUMA and NFAT and other transcriptional factors requires further study.

PUMA is partially controlled by P53, including the P53 transcriptional pathway in CA1 subregion neurons [102], P53-mediated cell death in a PD model [103], P53 and ERK1/2 pathways in SH-SY5Y neuroblastoma cells [65], and the NF-κB-P53-PUMA pathway in the rat hippocampus [61]. In addition, PUMA upregulation is dependent on ROS through the signaling cascade—ROS-JNK-P53-PUMA-caspase–3 and PI3K-AKT-FOXO3a-PUMA, which facilitates the occurrence and progress of Alzheimer’s disease (AD) [32]. PUMA upregulation is inhibited in copper/zinc-superoxide dismutase (SOD1)-over-expressing animals after transient global cerebral ischemia [102]. It is noteworthy that PUMA is significantly upregulated in motoneurons of SOD1^G93A^ mice with misfolded mutant SOD1 accumulated in the ER and an amyotrophic lateral sclerosis-like phenotype. The genetic deletion of PUMA significantly improves motoneuron survival and delays disease onset and motor dysfunction in SOD1^G93A^ mice [104].

P53 and other transcriptional factors can work together to trigger PUMA expression in the brain. Both P53 and PI3K-AKT-FOXO3a regulate PUMA expression in AD-related neurodegeneration [21]. JNKs can also phosphorylate and activate P53 on Ser15 and induce the transcription of pro-apoptotic target genes, such as PUMA, BAX (including JNK-P53-PUMA, JNK-AP-1[c-JUN]-PUMA) to mediate apoptosis in neurons [23, 59]. P73 (or P63)-mediated induction of pro-apoptotic genes SCOTIN and/or CHOP may contribute to PUMA-mediated apoptosis in cortical neurons [15]. P53, JNK/c-Jun, and PI3K-AKT-FOXO3a participate in the regulation of PUMA expression following Aβ exposure [21, 59, 100]. It should be noted that PUMA-deficient neurons are remarkably resistant to the induction of apoptosis and caspase activation in relation to diverse stimuli, including DNA damage, oxidative stress, ER stress, and proteasome inhibition [67, 105].

## Conclusions and perspectives

PUMA may be a critical regulator during ovarian development. Here, a model is proposed for PUMA-mediated apoptosis, which is regulated by transcriptional factors, a balance of survival and apoptotic factors, cell–cell interactions, and communications between the ER and mitochondria, especially in response to a variety of stimuli (Figs. 3 and 4). Knowledge of the specific and exact signals for PUMA in each pathway should be confirmed and improved. In particular, specific PUMAi, including P53 inhibitors, small molecules, ER stress inhibitors, and others, should be examined in terms of their physiology and pathology. PUMA inhibition may support therapy for human diseases and improve animal litter size.
